# Supplementary material for: Identification of Freezing-Responsive microRNAs and Their Targets in Chinese Jujube by Small RNA and Degradome Sequencing
Source: Int J Mol Sci. 2026 Sep 4;27(17):7912. doi: 10.3390/ijms27177912 (PMC13566890; doi:10.3390/ijms27177912)
Supplement: Supplementary file 1 [file ijms-27-07912-s001.zip › Supplementary Material S2/novel_miR_75.pdf]

Provisional ID : novel_miR_75
Score total : 0.5
Score for star read(s) : -1.3
Score for read counts : 0
Score for mfe : 0.2
Score for randfold : 1.6
Score for cons. seed :
Total read count : 54
Mature read count : 54
Loop read count : 0
Star read count : 0

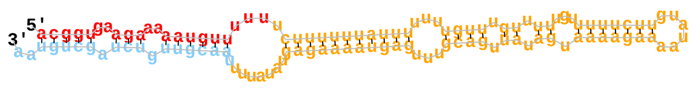

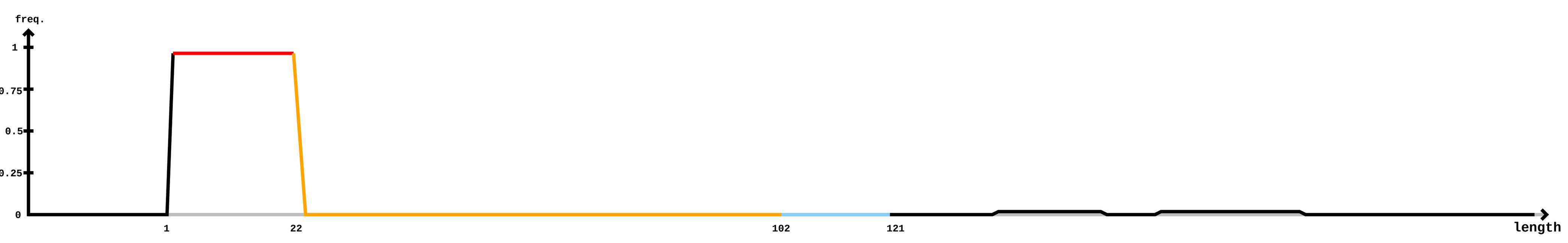

Mature Star

```
5'- ucaauuuuucaauuuuugcgguacggugaagaaaaauguuuuuucuuuuuuuauuuuuuuguuuguuuuuguuuuucuuguauaaaagaaaaugauauugacguuugaguaaaagaguauauuuuaacguugucuagcuguaagcacuauguauauuaaguuuauuuugaucuuugaucuuggagauuugauugaacuuugggcuaugagcgcacugugaaaguaaaggcuggcccacuacuugcuagca -3'   exp
    ...(((((((.....(((..((((((..(((..((((((....(((((((((((...((((.((.((...(((((((......))))))).)).)).))))...)))))))))))........)))))).))).)))))).)))..........((((((.((.(((((((......))))))).)).))))))((.(((...))).))...))))))))....((((((.........)))))).   reads   mm   sample
    ......................acggugaagaaaaauguuuuu........................................................................................................................................................................................................   26   0   LCK-1
    ......................acggugaagaaaaauguuuuu........................................................................................................................................................................................................   27   0   LCK-2
    ......................acggugaagaaaaauguuuuu........................................................................................................................................................................................................   1   0   LT-3
    ...............................................................................................................................................................Guuauuuugaucuuugau..................................................................................   1   1   LT-2
    ..........................................................................................................................................................................................uugauugaacuuugggcuaugagc.................................................   1   0   LT-3
```
